# Experimentally Validated Novel Inhibitors of *Helicobacter pylori* Phosphopantetheine Adenylyltransferase Discovered by Virtual High-Throughput Screening

**DOI:** 10.1371/journal.pone.0074271

**Published:** 2013-09-05

**Authors:** Chao-Sheng Cheng, Kai-Fan Jia, Ting Chen, Shun-Ya Chang, Ming-Shen Lin, Hsien-Sheng Yin

**Affiliations:** 1 Institute of Bioinformatics and Structural Biology and College of Life Sciences, National Tsing Hua University, Hsinchu, Taiwan; 2 TA Instruments-Waters LLC, Taipei, Taiwan; Institut Pasteur Paris, France

## Abstract

*Helicobacter pylori* is a major etiologic agent associated with the development and maintenance of human gastritis. The goal of this study was to develop novel antibiotics against *H. pylori*, and we thus targeted *H. pylori* phosphopantetheine adenylyltransferase (*Hp*PPAT). PPAT catalyzes the penultimate step in coenzyme A biosynthesis. Its inactivation effectively prevents bacterial viability, making it an attractive target for antibacterial drug discovery. We employed virtual high-throughput screening and the *Hp*PPAT crystal structure to identify compounds in the PubChem database that might act as inhibitors of *Hp*PPAT. d-amethopterin is a potential inhibitor for blocking *Hp*PPAT activity and suppressing *H. pylori* viability. Following treatment with d-amethopterin, *H. pylori* exhibited morphological characteristics associated with cell death. d-amethopterin is a mixed inhibitor of *Hp*PPAT activity; it simultaneously occupies the *Hp*PPAT 4'-phosphopantetheine- and ATP-binding sites. Its binding affinity is in the micromolar range, implying that it is sufficiently potent to serve as a lead compound in subsequent drug development. Characterization of the d-amethopterin and *Hp*PPAT interaction network in a docked model will allow us to initiate rational drug optimization to improve the inhibitory efficacy of d-amethopterin. We anticipate that novel, potent, and selective *Hp*PPAT inhibitors will emerge for the treatment of *H. pylori* infection.

## Introduction

More than 50% of the human population is infected with *Helicobacter pylori* [1,2], a pathogenic bacterium that causes chronic gastritis, peptic ulcers, and gastric carcinoma [3–5]. Various antibiotics, including amoxicillin, clarithromycin, and lansoprazole, have been used to treat *H. pylori* infection [6,7]; however, bacterial strains have often become resistant to these drugs, preventing effective treatment [8]. Thus, novel antibiotics against *H. pylori* are vital.

Coenzyme A (CoA) is an essential metabolic cofactor found in all living organisms, including bacteria, and is involved in many biosynthetic and degradative metabolic pathways, including the citric acid cycle and fatty-acid synthesis [9]. However, the bacterial enzyme phosphopantetheine adenylyltransferase (PPAT), which catalyzes the conversion of 4'-phosphopantetheine (Ppant) to 3'-dephospho-CoA in the penultimate step of CoA biosynthesis [10–13], shares an approximately 6% sequence identity with human PPAT [14,15]. Consequently, bacterial PPAT is an appropriate target for rational drug design [16].

Crystal structures of bacterial PPATs in both their free forms and complexed with various ligands are available [11,12,17–20]. PPAT has a homohexameric quaternary structure; each monomer contains 5 parallel β-strands and 6 α-helices that fold into a canonical dinucleotide-binding domain. Many of the residues involved in substrate binding are conserved, including Pro8–Thr10, His18, Lys42, Leu73, Leu74, Arg88, Arg91, Asp95, Tyr98, Glu99, Asn106, Ser129, and Ser130 [21]. An inhibitor of *Escherichia coli* PPAT (*Ec*PPAT) has been developed [22] that does not inhibit porcine PPAT, suggesting that the active-site residues of bacterial and mammalian PPATs differ substantially. Moreover, genetic footprinting studies have revealed that the inhibition of *Ec*PPAT prevents *E. coli* growth [16,23,24]; thus, bacterial PPAT has potential as an antibacterial target for drug discovery.

We recently reported the crystal structure of PPAT obtained from *H. pylori* (*Hp*PPAT, PDB ID: 3OTW) [21]. In our current study, we use virtual high-throughput screening (vHTS) with the *Hp*PPAT crystal structure, and compound structures retrieved from the PubChem compound database (http://pubchem.ncbi.nlm.nih.gov/ Accessed 2012) to identify novel inhibitors of *Hp*PPAT that could serve as lead compounds for the design of antibiotics that target *H. pylori* infection. The vHTS computational screening technique automatically and individually docks compounds from a specified database into the active site of a target protein, and estimates the binding affinity of the target protein toward the docked compound by using scoring functions [25–27]. Two docking programs, CDOCKER [28] and LigandFit [29], were used to screen a large number of compounds that are available in the PubChem compound database. The top ranked consensus compounds were then subjected to steady-state kinetic inhibition assays of the *Hp*PPAT-catalyzed forward reaction. These compounds were also incubated with *H. pylori* to characterize their antimicrobial activities. We used a steady-state kinetic inhibition assay and isothermal titration calorimetry (ITC) to characterize the d-amethopterin inhibition mechanism, the most effective overall inhibitor. Transmission electron microscopy (TEM) was performed to characterize the morphology of *H. pylori* after treatment with d-amethopterin. Finally, by examining the docked model of d-amethopterin and *Hp*PPAT, we were able to propose a d-amethopterin binding mode and identify important interactions between d-amethopterin and *Hp*PPAT, allowing us to rationally optimize the structure of d-amethopterin for the development of an antibiotic against *H. pylori*.

## Materials and Methods

### Materials

ATP, imidazole, Luria-Bertani broth, MgCl_2_, Tris-HCl, and NaCl were supplied by USB Corporation (Cleveland, OH). The compounds (omeprazole, T5568746, zanamivir, d-amethopterin, and AC1LDVWJ) used for inhibitory assays were purchased from Sigma-Aldrich (St. Louis, MO). Isopropyl β-d-1-thiogalactopyranoside and kanamycin were purchased from Protech (Taipei, Taiwan). Tris(2-carboxyethyl) phosphine hydrochloride was obtained from Acros Organics (Fair Lawn, NJ). Ppant was supplied by Enamine Ltd. (Kiev, Ukraine). All chemicals used were of analytical grade.

### Virtual high-throughput screening (vHTS)

The crystal structure of the *Hp*PPAT-CoA complex was retrieved from the Protein Data Bank (PDB code 3OTW) [21]. The complex has a hexameric structure; each *Hp*PPAT monomer binds one CoA molecule with the same orientation. Thus, *Hp*PPAT monomers could be used for vHTS. Prior to the docking study, the CoA molecule, water molecules, and sulphate were removed from the model. Explicit hydrogen atoms were added using Discovery Studio (DS) 2.0 software (Accelrys Inc., San Diego, CA). Protein atoms were typed according to the CHARMm force field [30]. Next, apo-*Hp*PPAT was energy minimized using the steepest descent algorithm available in DS 2.0 [31]. The compounds used were obtained. Using “enzyme inhibitor” as the query string, we retrieved 407 compounds for docking from the PubChem compound library (http://pubchem.ncbi.nlm.nih.gov/ Accessed 2012). All compounds were prepared using the Prepare Ligands protocol in DS 2.0.

The CDOCKER [28] and LigandFit [29] routines in DS 2.0 were used to find potential inhibitors of *Hp*PPAT. The docking parameters for the 2 programs were set to their default values. CDOCKER is a grid-based molecular dynamics docking algorithm that employs the CHARMm force field. The binding pocket was defined as a sphere with a radius of 15 Å, centered on the CoA molecule in the *Hp*PPAT-CoA complex, and encompassing the entire CoA structure. Ten random conformations for each compound were generated from the initial structure by using 1000 molecular dynamics steps at a temperature of 1000 K. Simulated annealing was then used to optimize the conformation of each compound at the *Hp*PPAT binding site, with 2000 heating steps and a target temperature of 700 K. The simulation used 5000 cooling steps to a target cooling temperature of 300 K. Refinement of the docked poses was performed using the full potential.

LigandFit is a shape-based method used to dock ligands into the active site of a protein. The *Hp*PPAT binding pocket was identified using the option “Find sites from Receptor Cavities” in DS 2.0. The active-site points were then manually edited to ensure that the CoA molecular structure would include these points. Monte Carlo simulations (15,000 trials) using a Class II Force Field [32] were employed to generate ligand poses. The grid extension was 3 Å, and the non-bonded cutoff distance was set to 10 Å with a distance-dependent dielectric constant. To avoid identical conformations, the root-mean-square-deviation cutoff was 1.5 Å, with a score threshold of 20 kcal/mol. A 1000-step rigid-body energy minimization was performed, and the top 10 docked conformations for each ligand were saved.

The scoring functions LigScore2 [33], PLP2 [34], Jain [35] and PMF [36] were used to evaluate and rank all docked ligand conformations. The steepest available descent algorithm was applied with the CHARMm force field to refine the best docked poses, using DS 2.0 and PyMOL (DeLano Scientific; http://www.pymol.org Accessed 2002.) [37] to visualize and inspect the docked poses. The *Hp*PPAT-compound interactions were identified using LIGPLOT v.4.0 [38].

### Protein Expression and Purification

Gene encoding for wild-type (WT) *Hp*PPAT was cloned, expressed, and purified as previously described [21]. The *E. coli* BL21(DE3) cells (Yeastern Biotech, Taipei, Taiwan) bearing a PET-28a(+) vector (Novagen, Whitehouse Station, NJ) that contained the WT *Hp*PPAT gene tagged at the N terminus with hexahistidine (His_6_) were inoculated into a 500 mL Luria-Bertani medium containing 30 mg/mL kanamycin, at 37 °C. When OD_600_ of the culture reached 0.7, isopropyl β-d-1-thiogalactopyranoside (final concentration, 1 mM) was added to the culture to induce protein expression. After 20 h of incubation at 20 °C, the cells were harvested by centrifugation using an Eppendorf 5810-R Centrifuge (Eppendorf, Hauppauge, NY) at 8000 × *g* for 20 min and 4 °C. The cell pellet was suspended in a solution of ice-cold Tris-HCl (20 mM) at pH 7.9, imidazole (80 mM), and NaCl (500 mM), and lysed on ice with a Misonix Sonicator 3000 (Misonix Inc., Farmingdale, NY).

The lysate was centrifuged at 7245 × *g* for 20 min at 4 °C, and the supernatant was applied to a 10 mL immobilized-Co^2+^ affinity column (BD Biosciences, Franklin Lakes, NJ), which had been pre-equilibrated with 20 mM Tris-HCl at pH 7.9, 100 mM imidazole, and 500 mM NaCl. After loading the lysate, the column was washed with the pre-equilibration buffer, and then the His_6_-tagged protein was eluted in a solution of 20 mM Tris-HCl at pH 7.9, containing imidazole (300 mM), and NaCl (500 mM). A Centricon Plus-20 centrifugal filter (Millipore, Billerica, MA) was used to remove the imidazole and to concentrate the protein. Purified *Hp*PPAT was characterized by SDS-PAGE (12% w/v acrylamide gel), and its concentration was determined using Bio-Rad Protein Assay kit reagents (Bio-Rad, Hercules, CA) and BSA as the standard.

### Quantitative *Hp*PPAT inhibition assay

For the inhibitory assay, we used EnzChek Pyrophosphate Assay kit reagents (E-6645, Invitrogen, Grand Island, NY) [39]. Various concentrations of tested compounds in H_2_O were added to reaction mixtures containing MgCl_2_ (6 mM), purine nucleoside phosphorylase (1 U/mL), inorganic pyrophosphatase (0.03 U/mL), 2-amino-6-mercapto-7-methylpurine ribonucleoside (0.2 mM), tris(2-carboxyethyl) phosphine hydrochloride (1 mM), and saturating concentrations of Ppant (60 µM) and ATP (3000 µM) with respect to the enzyme concentration. Afterward, *Hp*PPAT (final concentration, 25 nM) in a solution of 20 mM Tris-HCl at pH 7.9 and NaCl (125 mM) was added to each solution. The absorbance of each reaction was measured in a 1 cm path-length cuvette, at 360 nm for 120 s at 25 °C, using a Hitachi UV-visible U-3300 spectrophotometer. Residual activity was defined as the ratio of the turnover rate of *Hp*PPAT in the presence or absence of a test compound. Three independent assays were performed for each compound.

### Steady-state kinetic inhibition assay using d-Amethopterin

To determine the inhibition mode of d-amethopterin, a steady-state kinetic inhibition assay was performed using the procedure described in the preceding section, with the difference that the concentrations of Ppant and ATP were varied. The concentration of Ppant or ATP was fixed at a saturating concentration with respect to the enzyme concentration (25 µM and 1.6 mM, respectively), whereas the concentration of the other substrate was varied. The concentration of d-amethopterin in each reaction was 0 µM, 200 µM, or 250 µM. Each set of experimental data was fitted by nonlinear regression by using Prism 5 software (GraphPad Software Inc., La Jolla, CA) to determine the kinetic parameters. Assays were repeated at least 3 times, and the data points at each time point are reported as mean averages.

### Isothermal titration calorimetry (ITC)

To measure the binding affinity of *Hp*PPAT for d-amethopterin, we used a Nano ITC Low Volume system (TA Instruments–Waters LLC, New Castle, Delaware). The *Hp*PPAT (80 µM, 190 µl) in 20 mM Tris-HCl at pH 7.9 and 125 mM NaCl was titrated with d-amethopterin (608 µM, 50 µl), and the heat change for each titration was recorded at 25 °C. The mixtures were stirred at 300 rpm. Data were collected and analyzed using NanoAnalyze software (TA Instruments–Waters LLC). The titration curve was ﬁtted to the independent-site binding model.

### Antimicrobial assay

The antimicrobial activities of the tested compounds were determined as previously described [40]. In brief, *H. pylori* strain 26695 (1 × 10^7^ colony-forming units; ATCC#700392, Biosource Collection and Research Center, Hsinchu, Taiwan) was cultured in 3 mL *Brucella* Broth (Franklin Lakes, NJ) supplemented with 5% O_2_, 10% CO_2_, and 85% N_2_ (microaerophilic conditions) at 37 °C. After 24 h of incubation, each compound was added at 200 µM or 2000 µM to a culture and then incubated for 5 d. After incubation, OD_600_ was measured for each culture as an estimation of the antimicrobial activity of the compound. Three independent experiments were performed for each compound. In addition, TEM (JEM-1400 microscope; Jeol Ltd., Tokyo, Japan) was employed to characterize the *H. pylori* morphology at the completion of the d-amethopterin treatment.

### Dynamic light scattering

To examine whether the protein or compounds will precipitate, the dynamic light scattering (DLS) analysis was performed with ZetasizerNano S (Malvern Instruments; Spectris, Egham, UK). PPAT protein (4 µg/µl) and D-amethopterin (0.2 mM or 2 mM) in buffer (20 mM Tris, 125 mM NaCl, pH 7.9) were loaded in 1mm path length cuvette (Ratiolab^®^) and monitored at room temperature (25°C). All sample solutions were filtered through a membrane with 0.22 µm minisart filter.

## Results

### vHTS

To develop novel antibiotics against *H. pylori*, we screened 407 PubChem listed compounds against our recently reported crystal structure of *Hp*PPAT (PDB ID: 3OTW) [21] by using CDOCKER and LigandFit. Each retrieved compound was docked into the *Hp*PPAT binding site by using its top 10 energy-minimized conformations; the ranked lists of all docked poses were generated according to the appropriate scoring function; that is, DockScore for LigandFit or the CDOCKER interaction energy. We employed a set of scoring functions such as LigScore2, PLP2, Jain, and PMF to rescore and re-rank the docked poses. The 250 top-ranked docked poses according to each scoring function were retained and compared. If the same compound was found in at least 3 lists, it was retained for further analysis. We retained 89 LigandFit poses (31 compounds) and 173 CDOCKER poses (40 compounds). Compounds found by both programs were considered potential *Hp*PPAT inhibitors. We retained the 12 compounds with the highest scores from the 2 programs (Table 1). Table 1 lists these compounds with their PubChem compound ID, molecular weight, and rank according to the various scoring functions. These structurally and chemically diverse compounds could all be accommodated by the dinucleotide-binding site in *Hp*PPAT. The 3 compounds, **72440**, **24906324**, and **25200568**, share similar chemical structures, with **72440** having the greatest rank and highest score among the 12 compounds. In addition, the natural substrates of *Hp*PPAT (Ppant and ATP) and two *Ec*PPAT inhibitors (cpd**11** (7-iodo-pyrazoloquinolone) and cpd**12** (7-methylthio-pyrazoloquinolone) from the previous study [41]) were also included to do docking and score functions analysis. The scores of these compounds and that of **72440** were all listed in Table S1 in File S1. The **72440** compound displayed similar or higher scores than natural substrates and *Ec*PPAT inhibitors, suggesting that **72440** is a potential competitive inhibitor against *Hp*PPAT. Thus, we tested its inhibitory activity against *HpPPAT*. We also tested **4594**, **20112027**, **676113**, and **11946759**, which are commercially available and are readily soluble in water.

**Table 1:** Ranks for the Docked Poses of the 12 Best Compounds.

			**CDOCKER**				
**CID** ^a^	**Name**	**Mw**	**LigScore2**	–**PLP2**	**Jain**	–**PMF**	–**CIE** ^*a*^
**4594**	Omeprazole	345.4	122	249	240	-	-
**72440**	d-Amethopterin	454.4	37	167	3	122	3
**100450**	Lactoylglutathione lyase	388.4	68	210	72	221	14
**365754**	AC1L7RH4	418.6	124	-	111	-	32
**676113**	AC1LDVWJ	226.2	124	-	229	-	247
**5362033**	Enalaprilat	348.4	95	246	97	-	23
**11946759**	T5568746	368.5	173	-	83	237	-
**13216867**	SureCN9327107	416.5	53	172	6	141	4
**20112027**	Zanamivir	332.3	187	249	236	-	-
**24906324**	2qk8	452.4	56	187	14	200	6
**25200568**	CPD-6041	452.4	187	248	238	-	-
**25201794**	A835543	334.3	143	-	149	-	35
			**LigandFit**				
**CID**	**Name**	**Mw**	**LigScore2**	–**PLP2**	**Jain**	–**PMF**	**DockScore**
**4594**	Omeprazole	345.4	21	95	227	199	-
**72440**	d-Amethopterin	454.4	-	241	219	64	16
**100450**	Lactoylglutathione lyase	388.4	73	180	18	9	13
**365754**	AC1L7RH4	418.6	-	104	1	191	-
**676113**	AC1LDVWJ	226.2	65	1	149	-	-
**5362033**	Enalaprilat	348.4	129	194	8	163	-
**11946759**	T5568746	368.5	24	190	-	154	-
**13216867**	SureCN9327107	416.5	-	116	21	148	-
**20112027**	Zanamivir	332.3	7	-	99	38	12
**24906324**	2qk8	452.4	-	124	165	147	29
**25200568**	CPD-6041	452.4	-	182	26	73	18
**25201794**	A835543	334.3	-	233	249	219	-

The 250 top-ranked docked poses according to each scoring function are shown. Dash line means the ranks of this compound is out of 250.

^a^ CID, PubChem compound ID; CIE, CDOCKER interaction energy.

### *Hp*PPAT inhibitory assay

**Figure 1:**
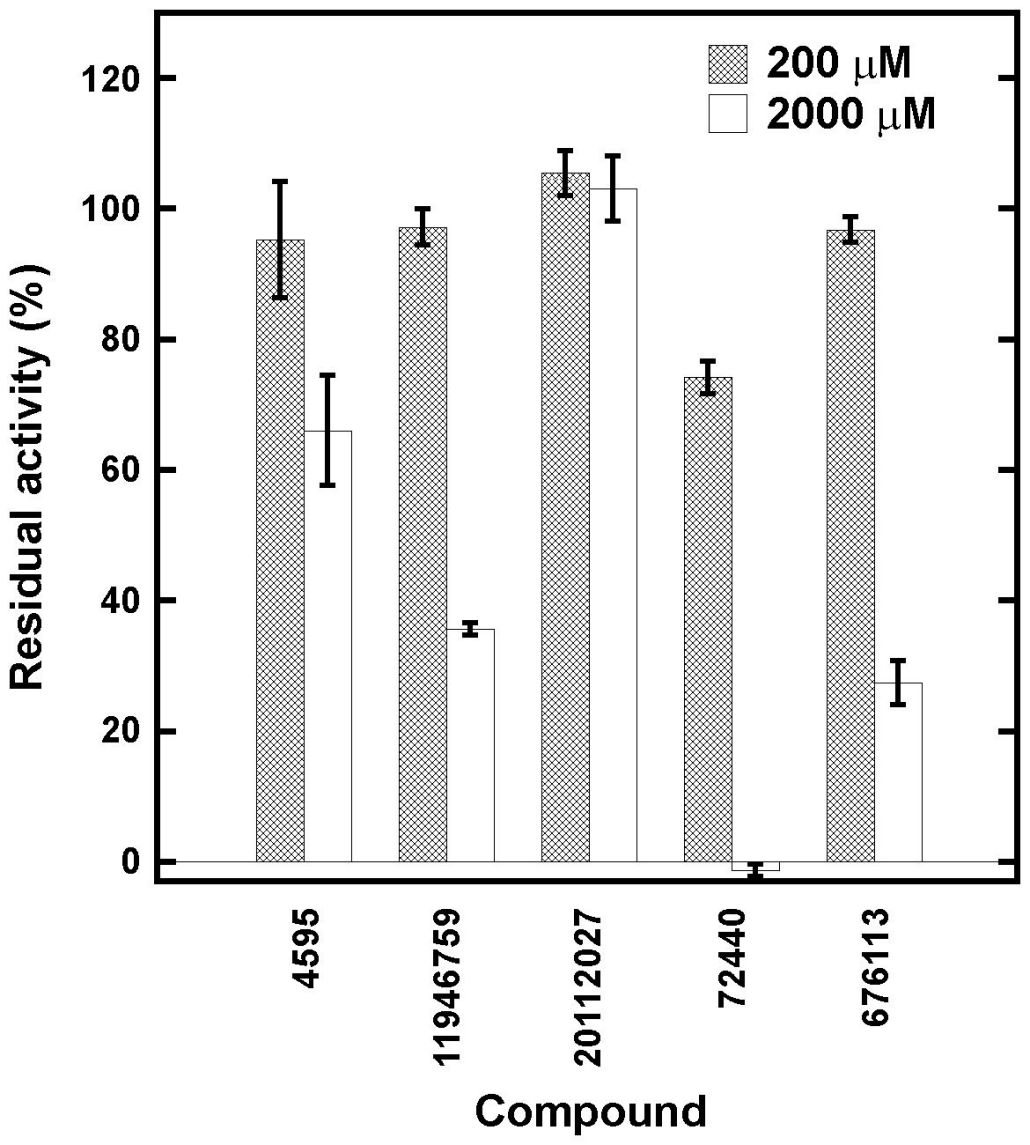
*Hp*PPAT inhibitory assay. The compounds were individually added to an *Hp*PPAT reaction solution to estimate the *Hp*PPAT inhibitory activity. Residual activity was defined as the ratio of the turnover rate of *Hp*PPAT before and after compound treatment.

With the exception of compound **20112027**, all aforementioned compounds provided approximately 40% or greater inhibition of *Hp*PPAT activity at 2000 µM (Figure 1). At this concentration, **72440** completely inhibited *Hp*PPAT activity, and at 200 µM it provided the greatest degree of inhibition among all of the candidate compounds (Figure 1), indicating that it is a potent inhibitor of *Hp*PPAT activity.

**Figure 2:**
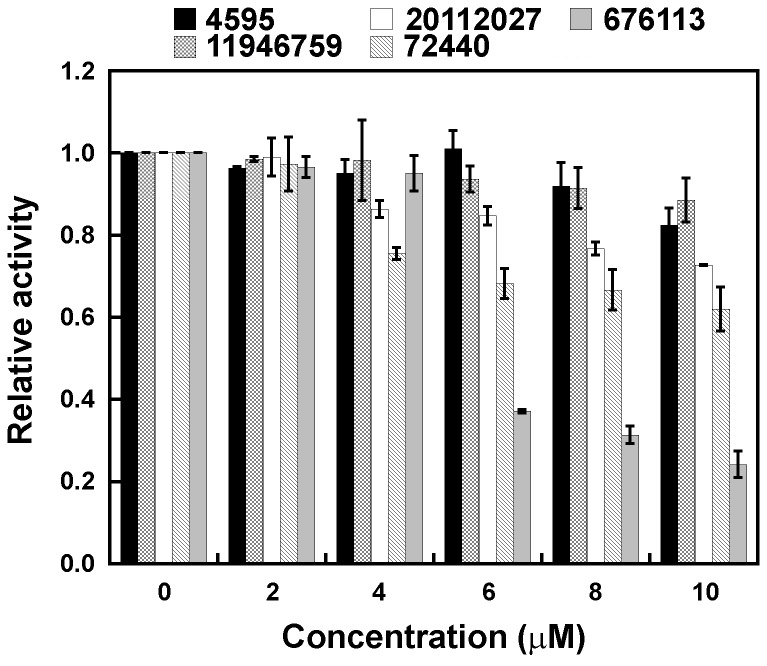
Antimicrobial activity. The *H. pylori* cells were cultured in the presence of various concentrations of the candidate compounds. Relative activity was defined as the ratio of the OD_600_ values before and after treatment with the compound.

**Antimicrobial assay. 4594**, **72440**, **676113**, **11946759**, and **20112027** were incubated with *H. pylori* to assess their antimicrobial activities [40]. The density of *H. pylori* cells (OD_600_) decreased significantly with an increasing concentration of **72440**, **676113**, or **20112027** (Figure 2), suggesting that these compounds suppress *H. pylori* viability. Even at the 2 greatest concentrations tested (8 µM and 10 µM), compounds **4594** and **11946759** did not significantly suppress *H. pylori* viability (Figure 2). Although **676113** exhibited the greatest antimicrobial activity, it was a weaker inhibitor of *Hp*PPAT activity than was **72440** (Figure 2), suggesting that **676113** may interfere with the functions of proteins other than *Hp*PPAT, which are required for *H. pylori* viability. Conversely, **72440** showed effective inhibition of both *Hp*PPAT activity and *H. pylori* viability.

### Steady-state inhibition assay

**Figure 3:**
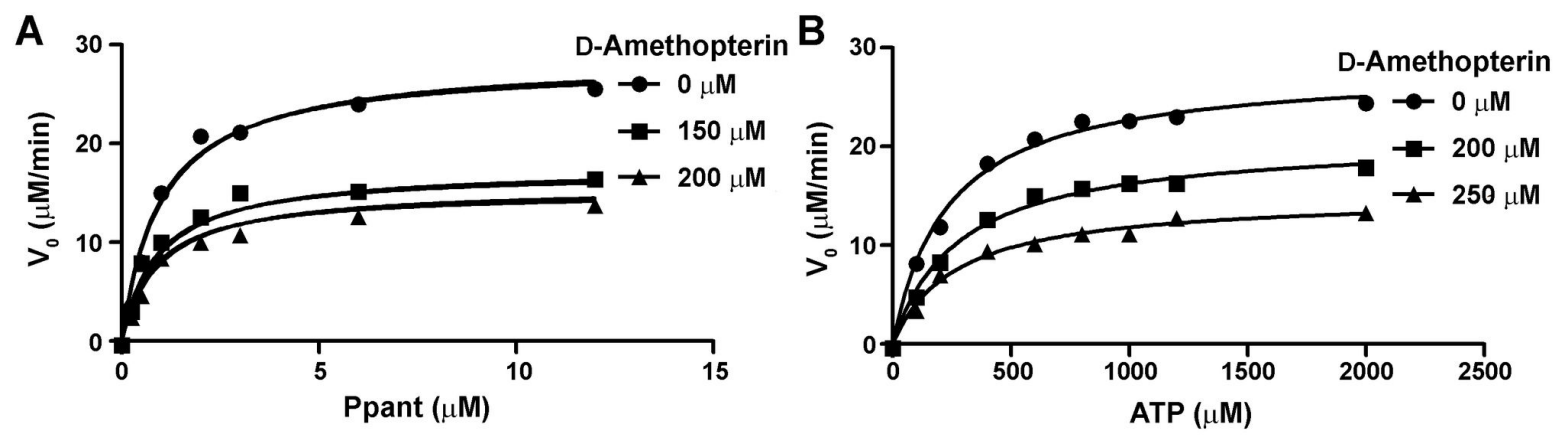
*Hp*PPAT inhibition by d-amethopterin. A steady-state kinetic inhibition assay was performed to determine the inhibition mode of d-amethopterin against *Hp*PPAT. The turnover rates of *Hp*PPAT were calculated for different concentrations of d-amethopterin. (A) Initial rates of the *Hp*PPAT-catalyzed reaction were obtained at different Ppant concentrations, while ATP was held at a saturating concentration. (B) Initial rates of the *Hp*PPAT-catalyzed reaction were measured at different ATP concentrations, while Ppant was held at a saturating concentration.

Compound **72440**, also known as d-amethopterin, inhibits *Pneumocystis carinii* dihydrofolate reductase activity [42]. To characterize how **72440** inhibits *Hp*PPAT activity, we performed a steady-state kinetic inhibition assay [41,43]. Various concentrations of d-amethopterin were incubated with *Hp*PPAT while holding the concentration of one of the substrates—ATP or Ppant—constant, and varying the other (Figure 3). Here, the dynamic light scattering test was performed to confirm d-amethopterin has no aggregation and precipitation. The dynamic light scattering data were shown in Figure S1 in File S1. The *Hp*PPAT turnover rate decreased with increasing d-amethopterin concentration when the Ppant concentration was varied and that of ATP (1.6 mM) was held constant (Figure 3A). Nonlinear regression curve fitting indicated that d-amethopterin is a mixed inhibitor of Ppant (*K*_i_ = 362.9 µM), suggesting that d-amethopterin binds to both free *Hp*PPAT and to the *Hp*PPAT-substrate complex [44]. Similarly, at a saturating concentration of Ppant (25 µM), d-amethopterin reduces the *Hp*PPAT turnover rate over a wide range of ATP concentrations (Figure 3B). d-Amethopterin also exhibits mixed inhibition against ATP (*K*_i_ = 267.4 µM).

### ITC

**Figure 4:**
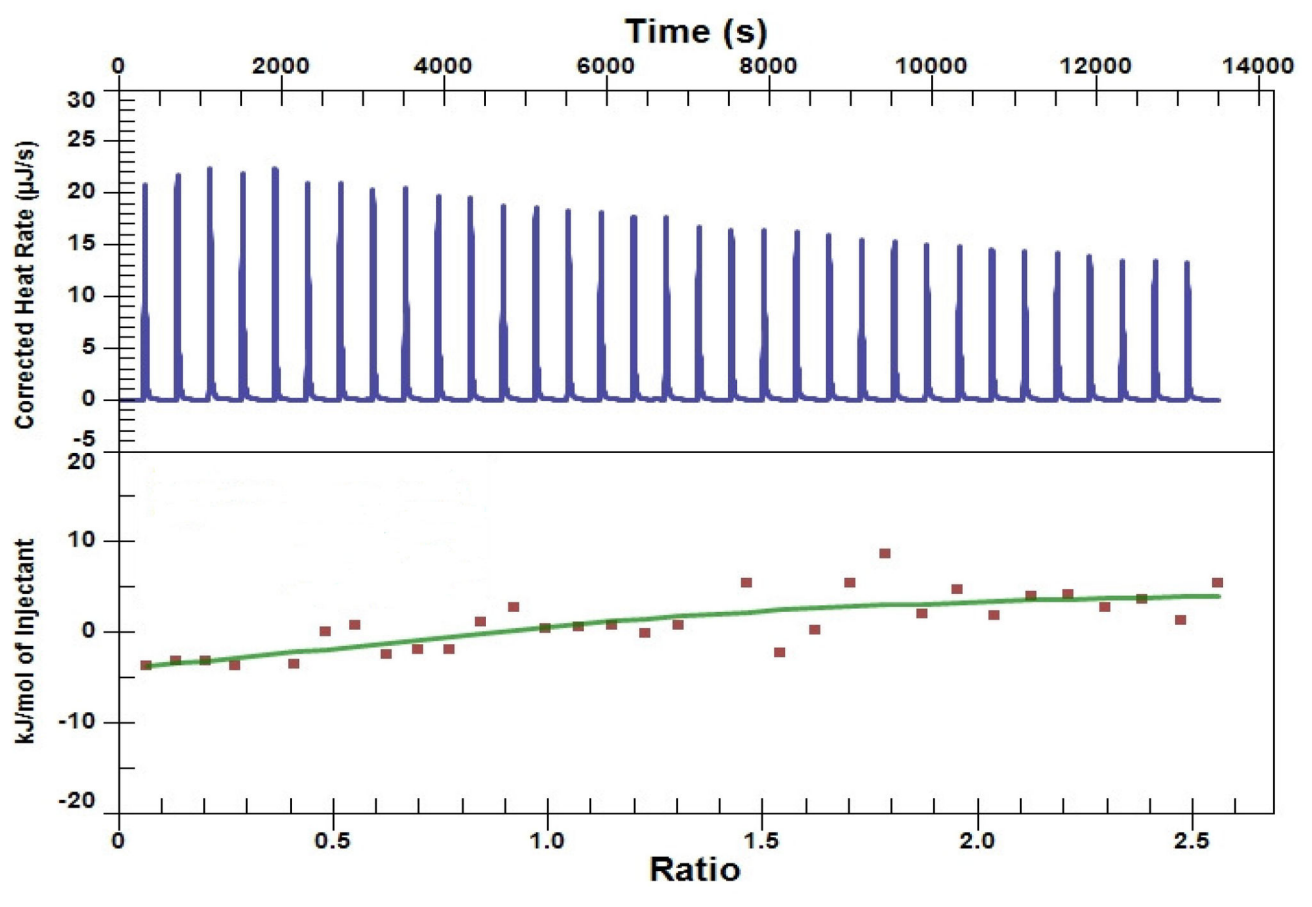
Isothermal titration calorimetry of the binding of d-amethopterin to *Hp*PPAT. When d-amethopterin was titrated into the *Hp*PPAT solution, the heat of binding was exothermic. The titration curve was fitted, assuming an independent-site binding model.

The binding affinity of *Hp*PPAT for d-amethopterin was determined by ITC. The binding of d-amethopterin is an exothermic process, and the binding heat gradually decreases with increasing d-amethopterin concentration (Figure 4). Fitting of the titration curve through several repeating, we obtained a *K*_d_ value of 31.16 ± 0.34 µM. The n value is 1.071 ± 0.211. The experimentally determined standard-state enthalpy (ΔH°) and the calculated standard-state entropy (ΔS°) for d-amethopterin binding were -12.10 ± 3.74 kJ/mol and 45.69 ± 1.23J/mol/K, respectively.

### Binding model

**Figure 5:**
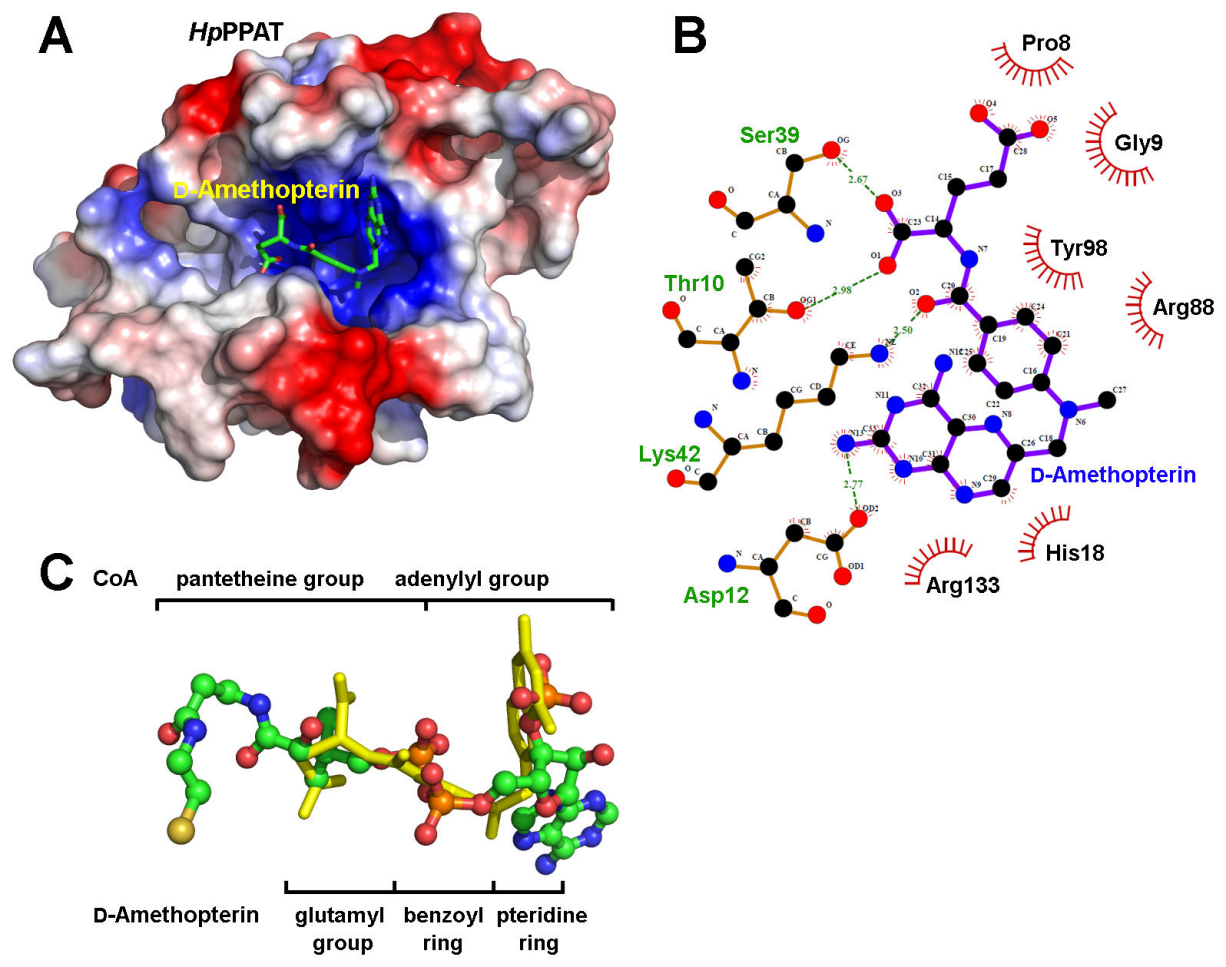
Model of *Hp*PPAT complexed with d-amethopterin. (A) d-Amethopterin lies in the dinucleotide-binding site of *Hp*PPAT. The *Hp*PPAT electrostatic potential surface is shown, and d-amethopterin is shown as a stick model. Positively and negatively charged surface regions of *Hp*PPAT are in blue and red, respectively. (B) Interactions between *Hp*PPAT and d-amethopterin according to LIGPLOT. Green dashed lines indicate hydrogen bonds. The *Hp*PPAT residues that participate in nonpolar interactions with d-amethopterin are represented as spoked arcs. (C) The structure of CoA in the crystal structure of the CoA-*Hp*PPAT complex (PDB code 3OTW) is superimposed onto that of d-amethopterin in the CDOCKER model. d-Amethopterin is shown as a yellow stick model, and CoA is shown as a ball-and-stick model.

The steady-state kinetic inhibition assay revealed that d-amethopterin is a mixed inhibitor against Ppant and ATP because d-amethopterin can simultaneously occupy the ATP- and Ppant-binding sites. Comparing the docked poses obtained using CDOCKER and LigandFit revealed that only the CDOCKER model predicts that d-amethopterin binds with both the ATP- and Ppant-binding sites (Figure 5A). The benzoyl and terminal pteridine rings in d-amethopterin occupy the ATP-binding site. In the LIGPLOT representation (Figure 5B), the side-chain oxygen atom in Asp12, hydrogen bonds with the amino group of the terminal pteridine ring on d-amethopterin. In addition, the C-terminal glutamate in d-amethopterin occupies a position that corresponds to the CoA pantetheine arm (Figure 5C), and hydrogen bonds with the side chains of 3 conserved *Hp*PPAT residues (Thr10, Ser39, and Lys42) (Figure 5B). We also found that 6 *Hp*PPAT residues (Pro8, Gly9, His18, Arg88, Tyr98, and Arg133) have nonpolar interactions with d-amethopterin (Figure 5B). With the exception of Arg133, these 6 residues are highly conserved in bacterial PPATs for substrate binding.

### Transmission electron microscopy (TEM)

**Figure 6:**
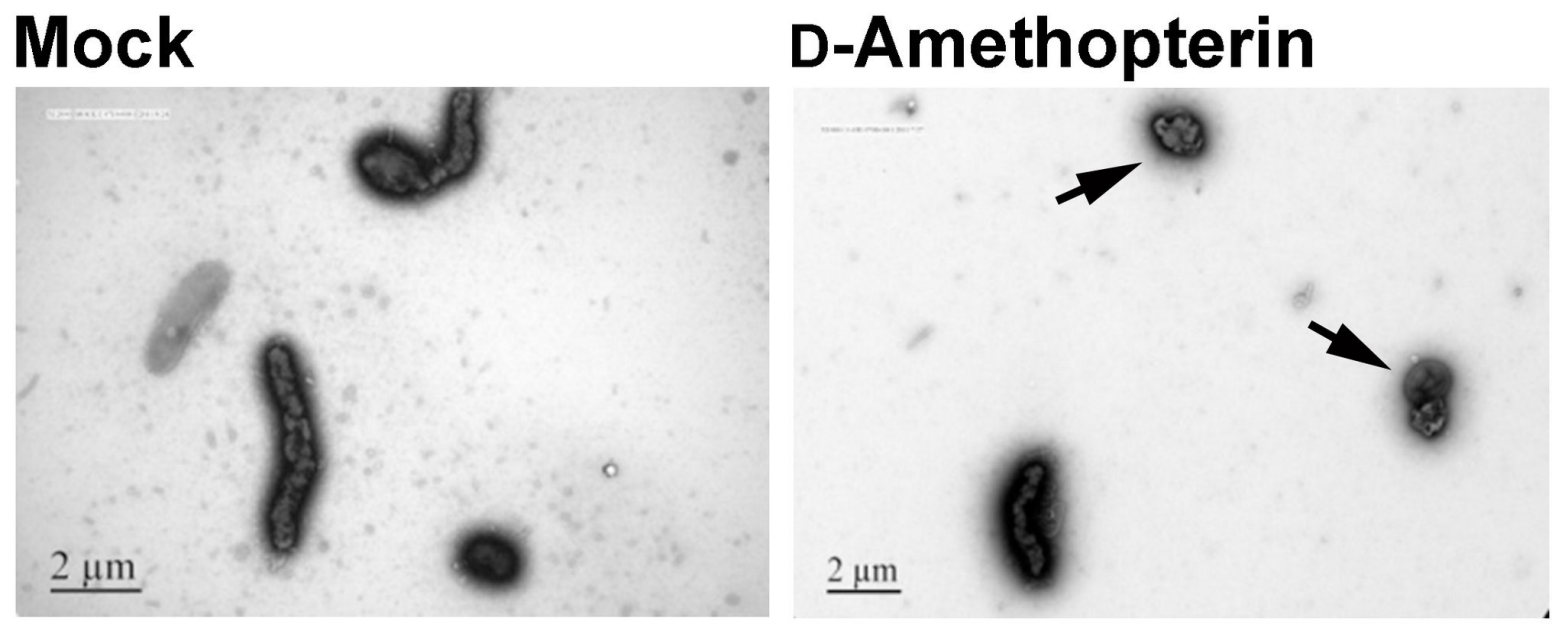
TEM images of *H. pylori* strain 26695. (A) *H. pylori* not treated with d-amethopterin. (B) *H. pylori* treated with d-amethopterin.

We used TEM to characterize the morphology of *H. pylori* after d-amethopterin treatment (Figure 6). The TEM image of untreated *H. pylori* showed that the cells had a normal helical bacillary appearance. However, *H. pylori* treated with d-amethopterin exhibited a coccoid morphology. It has been postulated that coccoid *H. pylori* cannot be cultured and that the coccoid shape is a morphologic manifestation of *H. pylori* death [45].

## Discussion

Using vHTS and the DS consensus scores facilitated the effective identification of potential inhibitors. Four of the 5 candidate compounds exhibited inhibitory activity against *Hp*PPAT. d-amethopterin in particular acted as an inhibitor of both *Hp*PPAT and *H. pylori* viability. Moreover, treatment with this mixed inhibitor converts the *H. pylori* morphology into a coccoid form, which is associated with *H. pylori* death. The CDOCKER docked pose for d-amethopterin received the highest score, and therefore, ranked first among the candidate compounds (Table 1), and this is consistent with our experimental results.

The steady-state kinetic inhibition assay revealed that d-amethopterin is a mixed inhibitor against Ppant and ATP, with micromolar *K*_i_ values (Figure 3). ITC data suggested that *K*_d_ also falls in the micromolar range, indicating that d-amethopterin is an appropriate lead compound for drug development.

Although d-amethopterin binds *Hp*PPAT, its binding enthalpy is small, as revealed by ITC measurements. Thus, the number of specific inhibitor–*Hp*PPAT interactions must be increased to enhance the inhibitory potency of d-amethopterin. Given our detailed binding model (Figure 5), we proposed that 2 functional groups in d-amethopterin require modification. The d-amethopterin benzoyl ring lies in a position that corresponds to the phosphate group in CoA. In the CoA-*Hp*PPAT complex, the Thr10, Lys42, Arg88, and Tyr98 side chains form hydrogen bonds with the CoA phosphate group [21]. However, in the CDOCKER model, only nonpolar interactions exist, and therefore, this ring structure should be modified to allow hydrogen bond formation. In addition, the C-terminal glutamate of d-amethopterin occupies only a part of the Ppant-binding site, suggesting that an extension of the d-amethopterin C-terminal region might be possible. In the crystal structure of the CoA-*Hp*PPAT complex, the CoA pantetheine arm makes nonpolar contacts with the conserved residues Pro8, Gly9, Ala37, Leu73, Leu74, and Asn106 [21]. Therefore, substituting additional nonpolar groups onto the d-amethopterin C-terminal region might increase the binding affinity of d-amethopterin toward *Hp*PPAT, thereby enhancing its inhibitory potency.

**Figure 7:**
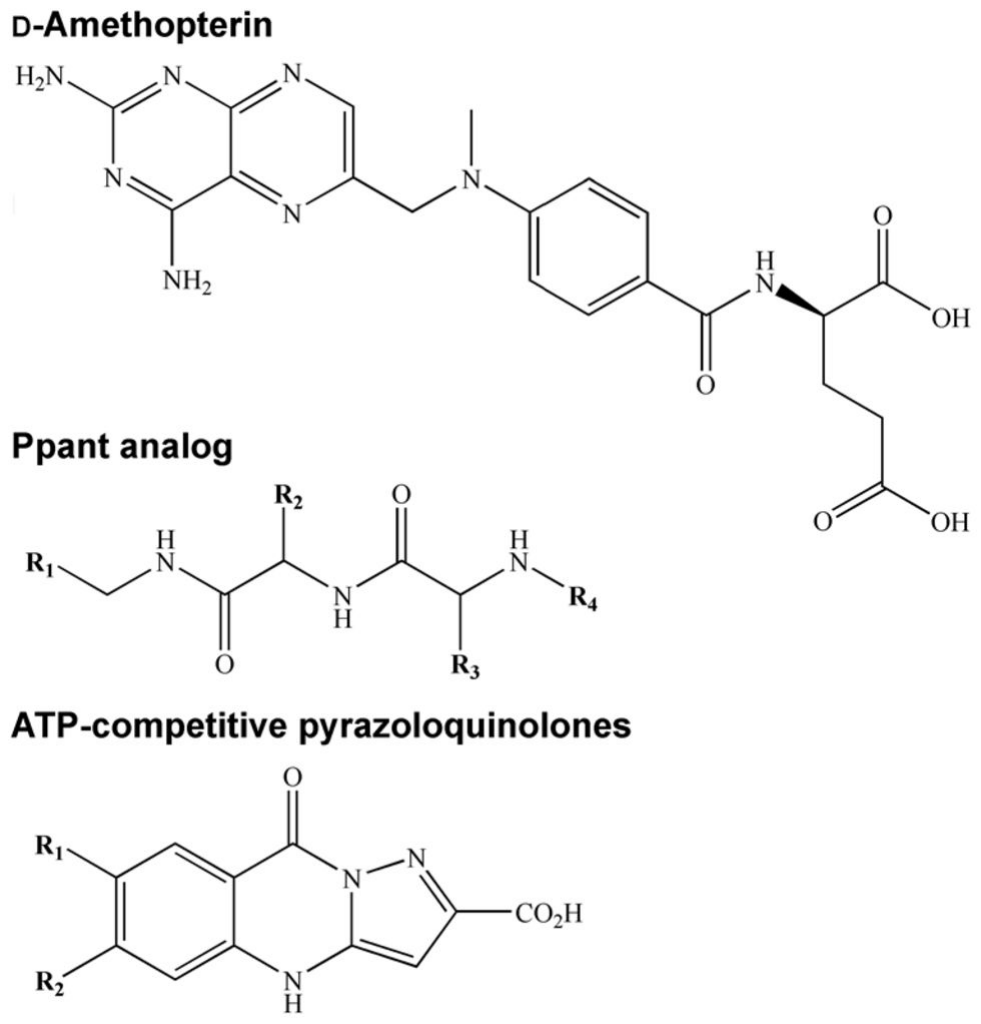
Structures of d-amethopterin and *Ec*PPAT inhibitors.

Finally, although a number of *Ec*PPAT inhibitors have been developed [22,41], their chemical structures are relatively different from that of d-amethopterin (Figure 7). The *Ec*PPAT inhibitors were designed using the Ppant structure as a template [22]; another class of inhibitors is the ATP-competitive pyrazolo-quinolone [41]. However, despite these inhibitors having significant inhibitory activities against *Ec*PPAT, they do not possess antibacterial activity. By contrast, d-amethopterin inhibits *Hp*PPAT-catalyzed reactions and suppresses *H. pylori* viability. d-amethopterin is an inhibitor of *P. carinii* dihydrofolate reductase (DHFR) [42], and has been used in cancer chemotherapy treatments, as an antibiotic, and as an antiprotozoal agent [46,47]. d-amethopterin acts as a folate antagonist for inhibiting DHFR activity. Structural [48] and mutagenesis [49] studies have revealed that carboxylic groups in d-amethopterin make significant contributions to hydrogen bonding and electrostatic interactions with DHFR. The C7 atom of the pteridine ring in d-amethopterin also participates in essential nonpolar contacts with DHFR. Therefore, these d-amethopterin functional groups must be modified so that DHFR and cancer-related effects are eliminated from the rational design of d-amethopterin. Accordingly, the results of our study suggest that d-amethopterin or rationally designed derivatives could be applied to other treatments, such as for *H. pylori* infection.

## Conclusions

In this study, vHTS was used to identify novel inhibitors of *Hp*PPAT. We screened 407 compounds by using CDOCKER and LigandFit, and identified d-amethopterin, which inhibits *Hp*PPAT activity and *H. pylori* viability, as a promising inhibitor. The *H. pylori* treated with d-amethopterin exhibits morphologic characteristics associated with cell death. This compound has sufficient inhibitory potency that it could be used as a lead compound for drug development. Our studies also revealed that the d-amethopterin binding affinity (*K*_d_) and inhibitory potency (*K*_i_) toward *Hp*PPAT are in the micromolar range, and that d-amethopterin acts as a mixed inhibitor in the suppression of *Hp*PPAT activity. d-Amethopterin can bind stably to the *Hp*PPAT active site by hydrogen bonding and nonpolar interactions. The conserved *Hp*PPAT residues Pro8–Thr10, Asp12, His18, Ser39, Lys42, Arg88, Tyr98, and Arg133 participate in these interactions. Moreover, on the basis of our binding model, we propose a rational drug optimization involving modification of the benzoyl ring and of the C-terminal region in d-amethopterin to increase d-amethopterin–*Hp*PPAT interactions and improve the inhibitory efficacy of D-amethopterin. We anticipate that novel, potent, and selective *Hp*PPAT inhibitors will emerge for the treatment of *H. pylori* infection because of this study.

## Supporting Information

File S1 **This file contains Table S1 and Figure S1.** Table S1, Scores for the Docked Poses of the d-Amethopterin, ATP, Ppant and two *Ec*PPAT inhibitors (7-iodo-pyrazoloquinolone (cpd11) and 7-methylthio-pyrazoloquinolone (cpd12) of [41]). Figure S1, Dynamic light scattering analysis of different concentrations of d-amethopterin and *Hp*PPAT.
(DOCX)
