# Supplementary material for: Experimentally Validated Novel Inhibitors of Helicobacter pylori Phosphopantetheine Adenylyltransferase Discovered by Virtual High-Throughput Screening
Source: PLoS One. 2013 Sep 5;8(9):e74271. doi: 10.1371/journal.pone.0074271 (PMC3764209; doi:10.1371/journal.pone.0074271)
Supplement: File S1 — This file contains Table S1 and Figure S1. Table S1, Scores for the Docked Poses of the d-Amethopterin, ATP, Ppant and two EcPPAT inhibitors (7-iodo-pyrazoloquinolone (cpd11) and 7-methylthio-pyrazoloquinolone (cpd12) of [41]). Figure S1, Dynamic light scattering analysis of different concentrations of d-amethopterin and HpPPAT. (DOCX) [file pone.0074271.s001.docx]

**Supporting Information**

**Table S1: Scores for the Docked Poses of the d-Amethopterin, ATP, Ppant and two *Ec*PPAT inhibitors (7-iodo-pyrazoloquinolone (cpd11) and 7-methylthio-pyrazoloquinolone (cpd12) of reference 41).**

|  | **CDOCKER** | | | | |
| --- | --- | --- | --- | --- | --- |
| **Name** | **LigScore2** | –**PLP2** | **Jain** | –**PMF** | –**CIE***^a^* |
| d-Amethopterin | 5.61 | 78.4 | 1.66 | 155 | 47.0 |
| ATP | 6.68 | 80.7 | 0.19 | 155 | 49.8 |
| Ppant | 5.22 | 63.8 | 1.56 | 90.1 | 41.0 |
| 7-iodo- pyrazoloquinolone | 4.65 | 45.5 | 0.21 | 79.2 | 29.4 |
| 7-methylthio- pyrazoloquinolone | 5.02 | 58.1 | 1.33 | 87.4 | 28.9 |
|  |  |  |  |  |  |
|  | **LigandFit** | | | | |
| **Name** | **LigScore2** | –**PLP2** | **Jain** | –**PMF** | **DockScore** |
| d-Amethopterin | 4.33 | 50.0 | -1.24 | 93.8 | 36.9 |
| ATP | 5.04 | 56.5 | -0.24 | 138 | 52.9 |
| Ppant | 4.86 | 43.5 | -1.48 | 99.4 | 39.4 |
| 7-iodo- pyrazoloquinolone | 2.56 | 34.4 | 1.95 | 59.9 | 14.9 |
| 7-methylthio- pyrazoloquinolone | 3.91 | 38.0 | -0.09 | 61.7 | 29.7 |

*^a^*CIE, CDOCKER interaction energy.

**Figure S1**


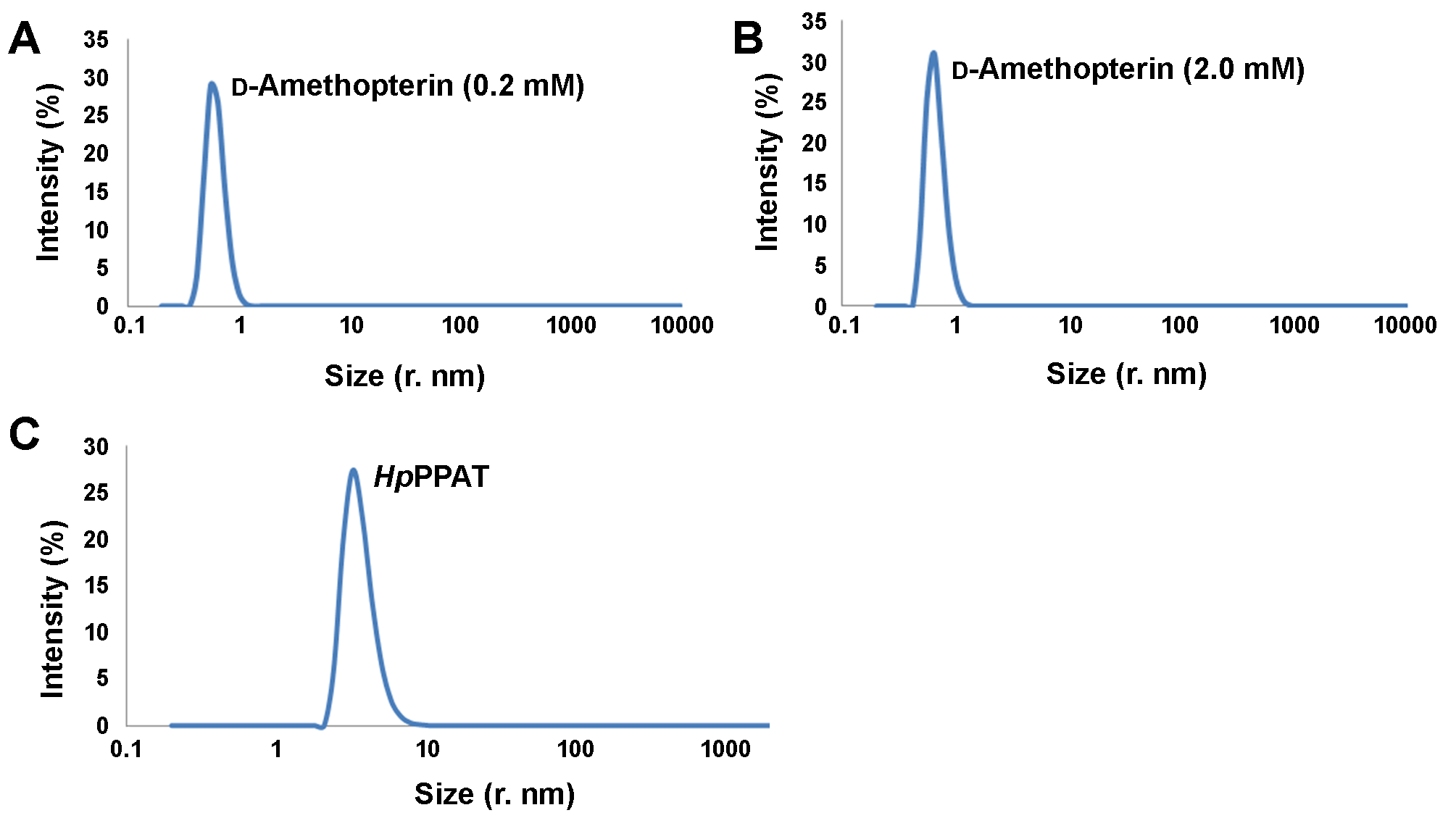


**Figure S1.** Dynamic light scattering analysis of different concentrations of d-amethopterin and *Hp*PPAT. (A) The average size of 0.2 mM d-amethopterin is 0.6862 nm. (B) The average size of 2 mM d-amethopterin is 0.7360 nm and (C) The average size of 4 μg/μl *Hp*PPAT protein is 5.529 nm. These results indicated that d-amethopterin compound and *Hp*PPAT protein have no aggregation in our assays.
